# A fast and reliable molecular method to detect Anisakidae in zooplanktonic Euphausiacea

**DOI:** 10.1051/parasite/2026038

**Published:** 2026-07-27

**Authors:** Anaïs Delhomme, Mélanie Gay, Alice Delegrange, Odile Bourgau, Maureen Duflot, Pierre Cresson

**Affiliations:** 1 ANSES, Laboratory for Food Safety F-62200 Boulogne-sur-Mer France; 2 Univ. Lille, CNRS, Université Littoral Côte d’Opale, IRD, UMR 8187 LOG- Laboratoire d’Océanologie et de Géosciences F-59000 Lille France; 3 IFREMER, Channel and North Sea Fisheries Research Unit 150 Quai Gambetta BP 699 F-62200 Boulogne-sur-Mer France

**Keywords:** Anisakidae, Euphausiacea, Zooplankton

## Abstract

Anisakidae is a family of marine nematodes, largely monitored in fish and cephalopods due to their zoonotic risk for human consumers. However, there is a need to further study the ecology of this family to better predict infection dynamics, particularly as global change affects marine ecosystem functioning generally, and specifically Anisakidae behaviour and infection levels, in line with the principles of the One Health approach. However, in-depth studies are hampered by technical difficulties in collecting data on members of the cycle that are not consumed by humans, namely zooplankton. This results in a glaring imbalance in terms of available data between fish and cephalopods on the one hand, and zooplankton on the other. Development of robust, rapid, and efficient methods for the detection of Anisakidae in zooplankton is therefore needed. Euphausiacea is considered to be the major zooplanktonic group hosting Anisakidae larvae. We therefore calibrated a PCR technique followed by Sanger sequencing to determine the presence of Anisakidae larvae in zooplanktonic Euphausiacea from the North Sea. This calibration was based in particular on the creation of artificially infested zooplanktonic samples, on which several molecular methods have been tested. When calibrated, we then applied this method to a small set of zooplanktonic samples and performed a preliminary parasitological survey. Prevalence values were higher (2.5% to 4.5%) than those reported in the literature, either due to the increased sensitivity of the molecular method and/or to changes in ecosystem functioning. This method has clear potential to push forward an integrated understanding of Anisakidae infection in zooplankton.

## Abstract (fr)

Les Anisakidae constituent une famille de nématodes marins, largement surveillés chez les poissons et les céphalopodes en raison du risque zoonotique qu’ils représentent pour l’homme. Néanmoins, une meilleure compréhension de l’écologie de cette famille est nécessaire pour une prédiction plus précise de la dynamique des infections, notamment face aux changements globaux qui affectent le fonctionnement des écosystèmes marins en général, et plus particulièrement le comportement et les niveaux d’infection par les Anisakidae, conformément aux objectifs de l’approche « Une seule santé ». Cependant, cette démarche est freinée par la difficulté technique de collecter des données sur les membres du cycle non consommés par l’homme, à savoir le zooplancton. Il en résulte un déséquilibre important dans les données disponibles entre les poissons et les céphalopodes d’une part, et le zooplancton d’autre part. Le développement de méthodes robustes, rapides et efficaces pour la détection des Anisakidae dans le zooplancton est donc indispensable. Les Euphausiacea sont considérés comme le principal groupe zooplanctonique hébergeant les larves d’Anisakidae. Nous avons ainsi calibré une méthode de PCR suivie d'un séquençage Sanger pour déterminer la présence de larves d'Anisakidae chez les euphausiacés zooplanctoniques de la mer du Nord. Cette calibration s'est notamment appuyée sur la création d'échantillons zooplanctoniques infestés artificiellement, sur lesquels plusieurs méthodes moléculaires ont été testées. Une fois calibrée, nous avons appliqué cette méthode à un petit ensemble d'échantillons zooplanctoniques et réalisé une étude parasitologique préliminaire. Les valeurs de prévalence étaient plus élevées (2,5 % à 4,5 %) que celles rapportées dans la littérature, probablement en raison de la sensibilité accrue de la méthode moléculaire et/ou de modifications du fonctionnement de l'écosystème. Cette méthode présente un potentiel certain pour faire progresser la compréhension intégrée de l'infestation par les Anisakidae chez le zooplancton.

## Introduction

Anisakidae Railliet & Henry, 1912 is a family of zoonotic parasitic nematodes that infect mainly marine animals [28]. This family includes genera such as *Anisakis, Contracaecum*, and *Phocanema* (ex-*Pseudoterranova* [3]), which are the most important in terms of zoonotic significance. All of these have received significant interest because they are ubiquitous parasites of marine fish species and because the consumption of raw or uncooked infected fish may generate hazards for human consumers [28]. The lifecycle of Anisakidae includes several hosts. Eggs are released into the environment with faeces of definitive hosts, cetaceans, pinnipeds, or piscivorous birds and hatch in the water. In the water column, larvae are consumed by zooplanktonic crustaceans. Zooplanktonic crustaceans are considered either paratenic or intermediate hosts (with development of the larva in the host), where larvae occur at L2 or L3 stages [25, 49]. Most frequently, fish and cephalopods are then infested, whether by consuming zooplankton, for zooplankton feeding species [7], or by consuming infested fish of lower trophic levels, for piscivorous fish species. Fish and cephalopods are mainly considered to be paratenic hosts, meaning that no morphological transformation of Anisakidae occurs in these species. The cycle is then completed when infested fish are preyed upon by a suitable definitive host, mainly cetaceans, pinnipeds, or piscivorous birds. Human infestation by Anisakidae occurs through the consumption of raw or undercooked infected seafood. Anisakidosis symptoms are mainly digestive or allergic. Most cases are observed in countries with high levels of fish consumption [45]. There is consequently a human-health concern related to increased consumption of infected and raw or undercooked fish products [9]. Due to this risk and given the need for infection data in fish to support risk assessment, documenting levels of infection by Anisakidae in fish has benefited from intense research activity. Similarly, as marine mammals are protected and, in some cases, endangered species, and as parasitism may be one of the potential causes of mortality (or a factor of morbidity for this group), parasites and specifically Anisakidae have also been screened for in stranded individuals [10, 26, 33], but with a lower effort than in fish.

Nonetheless, addressing the importance of the other members of the lifecycle, i.e. zooplanktonic crustaceans, is needed to better understand the ecological mechanisms underlying infection. In the context of implementation of the “One Health” approach, which calls for more integrated studies of zoonoses, understanding and predicting hazards related to the consumption of infected fish requires further study, and consideration of the other members of the cycle [9]. However, as illustrated by Poulin *et al.* [43], integrating parasitology into an ecological framework is a major challenge. Most studies are driven by human health related issues, and therefore focus on edible species in order to limit infection hazards for human consumers and overlook other members of the cycle [47]. Levels of Anisakidae infection in zooplankton have consequently been largely neglected, despite the importance of this group as a driver of fish infection and as a potential bottleneck in the cycle of Anisakidae [13, 32, 33, 42]. These studies mostly describe Euphausiacea as potential hosts of Anisakidae [15, 32, 49–51], even though Anisakidae larvae have also been observed in other groups, like Mysidacea and Copepoda. Ontogenic variation of the infection in jack mackerel *Trachurus trachurus* is, for example, directly related to ontogenic changes in diet, and the inclusion of Euphausiacea in the diet of larger fish individuals [7]. In the few studies focused on Anisakidae infection in Euphausiacea, prevalence values are generally low, and lower than 0.005%. Values ranging between 0 and 4% are nonetheless reported ([32], Table 1). A very peculiar case, with an extremely high prevalence of 40%, and “an exceptionally high rate of 78.0%” was recorded for *Anisakis simplex* larvae occurring in the Euphausiacea *Thysanoessa* spp. in the North Sea off Scotland and the Faroe Islands [50]. This value should, nevertheless, be considered with caution, particularly as the number of Euphausiacea individuals is not provided. But, as noted by Marcogliese [32], there is a major discrepancy in prevalence levels between zooplankton and fish, with levels being lower in zooplankton by several orders of magnitude. He highlighted this discrepancy as an interesting paradox that has to be elucidated in order to understand the ecological mechanisms of parasite transfer, and proposed hypotheses related either to massive consumption of zooplankton or to areas or periods where infection is favoured. The scarcity of data about Anisakidae infection in zooplankton did not allow any firm conclusions to be drawn.

**Table 1:** Existing data on the infection of zooplanktonic crustaceans by Anisakidae larvae. nd: no data provided in the original article.

Sampling area	Host	Number of analysed hosts	Number of infected hosts	Prevalence	Reference
North Pacific Ocean and Bering Sea	*Thysanoessa raschii* (Euphausiacea)	121	3	2.48%	[37]
	*Thysanoessa longipes* (Euphausiacea)	405	2	0.49%	
Northern North Sea, northern Scotland and the Faroe Islands	*Thysanoessa inermis* (Euphausiacea)	1 335	18	1.35%	[48]
	*Thysanoessa longicaudata* (Euphausiacea)	950	3	0.003%	
Northeast Atlantic Ocean	*Mesopodopsis slabberi* (Mysidacea)	131	1	0.763%	[31]
Northeast Atlantic Ocean and northern North Sea	Euphausiacea	nd	nd	0 to 4%; a peak at 78%	[50]
Northern North Sea	*Paraeuchaeta norvegica* (Copepoda)	1 955	5	0.26%	[24]
Pacific Ocean, Prince William Sound, Alaska	*Thysanoessa raschii* (Euphausiacea)	10 427	2	0.019%	[51]
	*Euphausia pacifica* (Euphausiacea)	7 443	1	0.013%	
Atlantic Ocean, Estuary Ria de Vigo, Spain	*Nyctiphanes couchii* (Euphausiacea)	155 775	3	0.0019%	[15]
	Unidentified Mysidacea	nd	1	nd	
Pacific Ocean, Baja California peninsula, Mexico	*Nyctiphanes simplex*, (Euphausiacea)	176 to 1 330	2 to 6	0.005 to 0.094%	[13]

One of the major challenges in determining Anisakidae infection levels in zooplankton is largely technical. The studies previously cited are based on visual observation of freshly collected samples of zooplankton, whether through binoculars or microscopes, and followed by zooplankton dissection and molecular identification of the parasite species, for the most recent paper [15]. This approach is associated with several drawbacks. Firstly, sorting, identifying, and observing numerous zooplankton individuals is time-consuming. This is a major issue if observations are to be generalised, over large spatial or temporal scales, as the low prevalence requires the collection of several hundred or even thousands of zooplankton individuals to gain a robust picture of infection patterns (Table 1). In addition, visual observation has to be performed immediately after collection. Most zooplankton preservation methods, whether freezing or chemical fixation with formalin-derived solutions, make them opaque and the larvae can no longer be seen through them [13, 50]. These types of observations are, therefore, limited to coastal sampling, where it is possible to return to the laboratory on land immediately after sampling. For samples taken during offshore campaigns, microscopic observation on board can be complicated by rough sea conditions. In addition, zooplankton is not the focus group of most surveys, usually dedicated to the collection of data about fish to ensure fisheries sustainability. Furthermore, sea campaigns are rare opportunities. They often involve a great deal of work, carried out by small teams. As a result, it is usually not possible to devote a significant amount of time to ancillary work on zooplankton, as time between two plankton-sampling operations is limited. Progressing towards more exhaustive investigation of Anisakidae levels in zooplankton therefore requires new methodological developments [32].

Furthermore, the hazards associated with eating infected fish have led to the development and standardisation of robust methods for collecting, detecting, and identifying Anisakidae larvae in fish. Fish muscle is either crushed under a hydraulic press, and then observed under UV light [20] or dissolved in pepsin solution [30]. Then, after preliminary identification based on morphological features, quantitative or conventional PCR followed by Sanger analysis allows for molecular identification of the larvae to the species level [11, 38, 46].

In this context, the aim of the present study was to adapt the molecular method developed to detect Anisakidae in fish for Euphausiacea. Numerous constraints, such as the relative size between Euphausiacea and the parasite inside, the impossibility of knowing whether there will be UV fluorescence, the opacity of the Euphausiacea or the difference in matrix, preclude direct application of the methods used in fish to Euphausiacea. It was therefore necessary to optimise all the steps to produce a protocol with sufficiently low sensitivity and high specificity for the detection of Anisakidae. The method also has to be simple to use and fast enough to process a large number of samples, as required by the expected low prevalence, and in order to apply the method over large spatial and temporal scales. Development was carried out in two steps. As it was impossible to know *a priori* whether Euphausiacea samples were infected, samples containing Euphausiacea and Anisakidae were artificially created first, to test and adapt the method originally created for fish. The method was then applied to real samples collected during a survey in the North Sea. This preliminary parasitological study provided some initial results on the levels of infection in Euphausiacea, in the North Sea.

## Material and methods

### Field sampling

**Figure 1:**
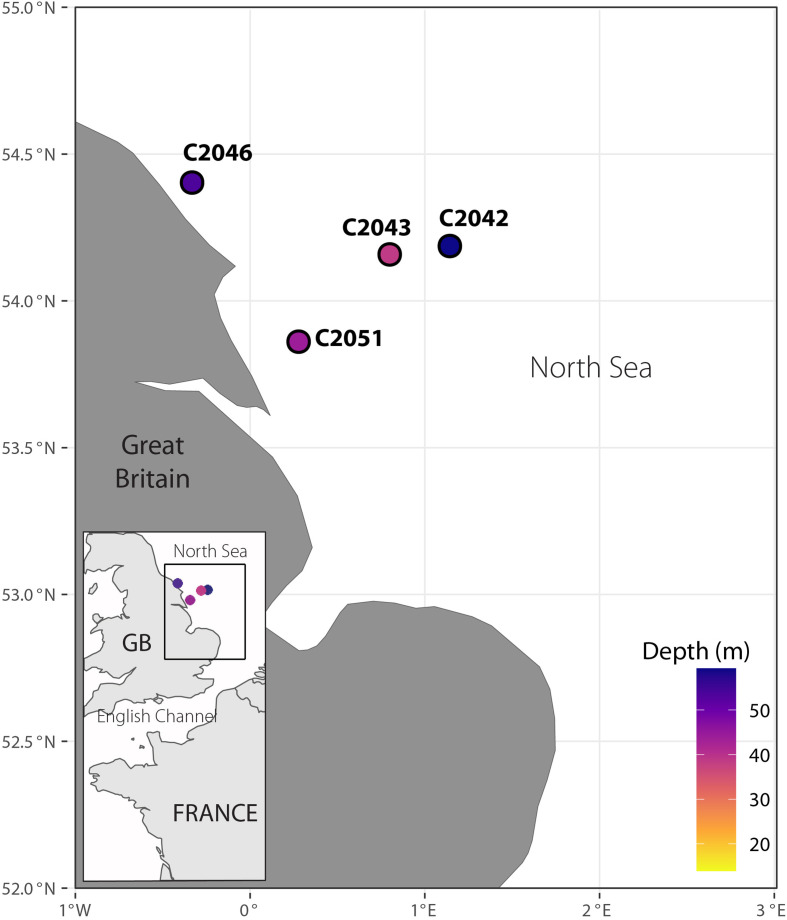
Map of stations sampled during the IBTS 2024 survey. The general location of sampling is presented in the bottom left inset, in which the rectangle highlights the more precise location of the sampling stations. Euphausiacea individuals from station C2043 (in italics) were used to calibrate the method, which was then applied to samples from the other three stations. Depth of the stations is illustrated by the colour of the dots.

Zooplankton samples were collected at 4 stations in the North Sea during the International Bottom Trawl Survey (IBTS) in January and February 2024 [2] (Fig. 1). IBTS is an international survey aimed at informing fisheries management by producing indices of abundance for the exploited fish species. Additionally, it provides an integrated picture of the ecosystem, by sampling all of its components, from plankton to top predators. Evaluation of commercial fish species also includes collection of their larvae. This sampling was used to opportunistically collect zooplankton occurring with fish larvae using a Methot Isaac Kidd (MIK) net. MIK is a 13 m long, 2 m diameter cylindroconic net, of 1.6 mm mesh size, narrowing to 500 μm in the final metre. The net was hauled at night during a double oblique tow from 5 m above the sea floor up to the surface, for at least 15 min. After hauling, samples were quickly processed on board, to discard all non-target organisms (e.g. gelatinous organisms and fish larvae) then stored frozen (−20 °C).

### Sample preparation

Back in the laboratory, and after defrosting, samples were carefully processed to select Euphausiacea individuals only, on the basis on specific morphological parameters [16]. Identification to the species level was not possible, as the conservation of Euphausiacea in sea water and thawing led to the detachment of the thoracic limbs, which are mandatory parts for identification.

Euphausiacea from station C2043 were used for method development because it was thought on board that this was the station where they were most abundant, and considering *a priori* that the repeated steps of method calibration would require a large number of samples. Then, Euphausiacea individuals from stations C2042, C2046, and C2051 were used for method application and the determination of infection levels. Euphausiacea individuals from all four stations were separated individually and frozen again.

The feasibility of the UV light detection method was tested by applying the same approach as for fish: all Euphausiacea individuals from stations C2042, C2046, and C2051 were observed under UV light (366 nm), following the standardised method ISO 23036-1:2021(F) [17]. Because of their thinness, the Euphausiacea were not crushed by the press. Once the method was calibrated, Euphausiacea were analysed individually with the developed method for the parasitological study. At each station, 198 Euphausiacea were analysed, leading to a total number of samples analysed of 594 over the three stations.

### DNA extraction

Prior to the experiment *per se*, a subset of Euphausiacea and Anisakidae L3 larvae were individually weighed, to estimate the average mass ratio between one Anisakidae individual and one Euphausiacea individual. One Euphausiacea and half of an Anisakidae L3 larvae were weighed using a precision scale, (to the nearest 10^−3^ mg), considering that in a Euphausiacea, the Anisakidae should be at most half the size of a L3 larvae. Assessing these mean weights was necessary to set up artificial samples with a relative importance of Euphausiacea and Anisakidae consistent with natural mass ratios (Table 2).

**Table 2:** Mass of Anisakidae and Euphausiacea for each sample used for method calibration. Samples with 0% and 100% proportion of Anisakidae are negative and positive controls, respectively and are presented for information purposes only.

Anisakidae proportion	Anisakidae mass (mg)	Euphausiacea mass (mg)
0%	0	150
100%	20	0
1%	2	198
0.5%	2	398
0.25%	2	796
0.1%	2	1 980

For method development, Euphausiacea from station C2043 and *Anisakis* individuals (L3 larvae) collected in the visceral cavity of hake *Merluccius merluccius* caught in the Celtic Sea (FAO Zone 27.VII.h) in a previous project were used. Euphausiacea and *Anisakis* were separately ground using a Mixer Mill MM 400 (Retsch GmbH, Haan, Germany) at 25 Hz for 2 min, with six 7 mm diameter stainless steel beads in a grinding bowl. Then, shredded Anisakidae and Euphausiacea were artificially mixed. The term “artificially” is used to refer to samples prepared by mixing Euphausiacea and *Anisakis* larvae in different proportions. The ratio of 1% was considered to be a representative value for the average mass proportion between Anisakidae and Euphausiacea. For the parasitological study, Euphausiacea from stations C2042, C2046, and C2051 were ground with two 3 mm diameter beads, following the same parameters as above.

Artificial samples of Euphausiacea with Anisakidae were used to compare the performance of four DNA extraction methods: proteinase K internal protocol (named proteinase K), a Promega Wizard Genomic DNA purification kit (Promega, Madison, WI, USA), a QIAGEN Genomic-tip kit (QIAGEN, Hilden, Germany), and a Nucleobond Column AXG kit (Macherey-Nagel, Düren, Germany).

Efficiency of the four extraction methods was tested using artificial Anisakidae/Euphausiacea samples of 1% and 0.5% proportions. Then, the proportion of Anisakidae in the artificial sample was decreased (0.25% and 0.1%) to test the selected method’s detection limits.

For the first method, samples were lysed using proteinase K at 20 mg mL^−1^ (QIAGEN) and 10× lysis buffer (60 mg L^−1^ Tween 20, 0.5 M EDTA and Trisbase) for 6 h at 55 °C, at 600 rpm in a Thermomixer C. Then, the proteinase K was inactivated for 10 min at 95 °C, at 300 rpm. After brief centrifugation, supernatant was collected and preserved. All three commercial kits were applied, following the manufacturer’s instructions. Two control solutions, one positive with only Anisakidae tissue, one negative with only Euphausiacea tissues, were also tested. DNA extractions were performed in triplicate.

### PCR amplification and electrophoresis

For all samples (whether artificially created or originating from natural samples), a fragment of 530 bp of the mitochondrial cytochrome *c* oxidase subunit II (*mtDNA Cox2*) gene was amplified using the primers F-univ-nem (5′– GGT GTT CTT TCT TTT GTT TCT G –3′) and R-univ-nem (5′– ATA AAA CTA TGG TTA GCC CCA C –3′), according to Seesao *et al.* [46]. These primers are specific to *mtDNA Cox2* from Anisakidae and *Hysterothylacium*, formerly belonging to the Anisakidae family but now included in the Raphidascarididae family [52]. PCR reactions were carried out in a total volume of 50 μL with 2 μL of extracted DNA and 48 μL of PCR mix consisting of 10× reaction buffer, 25 mM MgCl_2_, 10 mM dNTPs, 50 μM of each primer (Eurofins, Nantes, France) and 5 U/μL of HotStarTaq DNA polymerase (QIAGEN, Germany).

Amplifications were carried out in a 2720 Therma Cycler (Applied Biosystems, Foster City, CA, USA) with a preheating step at 95 °C for 15 min, 50 cycles of amplification (95 °C for 30 s, 58 °C for 30 s, 72 °C for 30 s) and a final extension step at 72 °C for 7 min. All PCR products were visualised on a 1% agarose gel with 3.5‰ of ethidium bromide for visualisation.

### Result analyses and parasitological descriptors

Each DNA extract was tested in triplicate by PCR. The method was validated (i.e. considered reproducible) if, for each proportion, (i) at least two amplifications were positive, (ii) if controls displayed expected results, i.e. amplification for positive control and no amplification for negative control, and (iii) in the absence of false positive or negative. For the parasitological study, each extracted sample was analysed three times by PCR. It was considered positive if DNA amplification was observed after PCR on the electrophoresis gel for at least two out of three times for one sample, and if controls displayed expected results as mentioned above.

Euphausiacea infection was expressed as prevalence, i.e. as the numbers of individuals where Anisakidae were detected divided by the total number of individuals analysed [6].

In the parasitological study, in order to determine the parasite species observed in Euphausiacea, PCR products of positive samples and 530 bp were sequenced from both sides (forward and reverse) using Sanger sequencing (Eurofins). The obtained sequences were analysed by GenBank BLAST software (version 2023.2.1.0).

## Results

### Selection of the DNA extraction method

**Figure 2:**
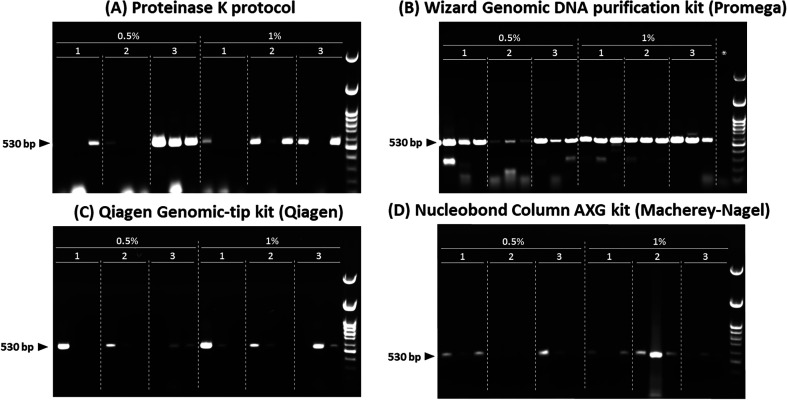
Agarose gel electrophoresis of PCR products from DNA extracted from 0.5 and 1% Anisakidae/Euphausiacea mass ratio samples after (A) proteinase K protocol, (B) Wizard Genomic DNA purification kit, (C) QIAGEN Genomic-tip kit, and (D) Nucleobond Column AXG kit. Each condition (method/mass ratio) was extracted three times (numbered 1, 2, and 3). Each sample was amplified and deposited in triplicate. Water was used as the negative control (*).

Using solutions with 0.5% and 1% Anisakidae/Euphausiacea mass ratios, reproductive results were obtained with a Wizard Genomic DNA purification kit only, without any false positives or negatives (Fig. 2). Results obtained with the other three methods were inconclusive, and provided inconsistent results, such as no effect of increased concentration.

Consequently, the Promega kit was used on artificial samples of Euphausiacea with proportions of 0.25% and 0.1% of Anisakidae for further validation. Again, each proportion was extracted three times and each sample was amplified three times. At least two replicates out of three for each proportion were positive.

### Parasitological study

Application of DNA extraction with the Wizard Genomic DNA purification kit to Euphausiacea from stations C2042, C2046, and C2051 detected 30 positive samples out of 594 Euphausiacea individuals.

Sequencing of positive samples made it possible to identify 22 samples as *Anisakis simplex*, *A. pegreffii*, and *Hysterothylacium aduncum* (Table 3). Of note, *A. simplex* was the most abundant parasite, with a proportion of 82% among all parasites. Seven samples from station C2046 and one from station C2051 could not be identified with sequencing. Either sequencing failed or obtained sequences were not analysable. Thus, prevalences were calculated from the number of infected Euphausiacea confirmed by Sanger sequencing. They ranged from 2.5 to 4.5%.

**Table 3:** Anisakidae prevalence at the three stations C2042, C2046, and C2051 of the IBTS 2024 survey and results of species identification by sequencing. Number of positive Euphausiacea: number of Euphausiacea samples with positive results on PCR detection of Anisakidae. Parasite identification: results of species identification by Sanger sequencing.

Sampling station	Depth (m)	Number of Euphausiacea	Number of PCR-positive Euphausiacea	Number of identified parasite species	Parasite identification	Prevalence (%)
C2042	58.96	198	9	9	6 *Anisakis simplex*	4.5%
					2 *Hysterothylacium aduncum*	
					1 *Anisakis pegreffii*	
C2046	53.52	198	15	8	7 *Anisakis simplex*	4.0%
					1 *Hysterothylacium aduncum*	
					7 individuals not identified	
C2051	43.98	198	6	5	5 *Anisakis simplex*	2.5%
					1 individual not identified	

The standard UV light method provided inconclusive results: fluorescence was observed on the cephalothorax of some Euphausiacea; PCR was negative for these samples. In addition, no fluorescence was observed for samples with positive PCR results.

## Discussion

### Method development

The first objective of this study was to develop and validate a fast and reliable method to detect Anisakidae larvae in zooplankton and more specifically in Euphausiacea. Importantly, former studies on the presence of Anisakidae in zooplanktonic crustaceans relied on visual observations. However, this method is time-consuming and requires working on fresh material since crustaceans rapidly become opaque after death, reducing the ability to see the Anisakidae larvae within the crustaceans.

Following former studies on the fluorescence of Anisakidae larvae in fish [20, 40], a similar approach was adopted. However, the results based on fluorescence and molecular analysis were inconsistent, both because the fluorescence observed in some individuals could not be correlated with the detection of Anisakidae, and because PCR-positive individuals did not fluoresce (data not shown). Nonetheless, even though inconclusive, these results must be published and discussed, to avoid repeating them, and to challenge the limitations of the UV light method, consistently with the importance of publishing negative results [4]. Fluorescence-based detection of Anisakidae relies on the presence of lipofuscin in the parasite, a molecule that accumulates in tissues with parasite’s age and becomes fluorescent under UV light [40]. It can, therefore, be hypothesised that lipofuscin is absent or present in low concentrations in L2 larvae, which prevents the observation of fluorescence at this stage, and accumulates afterwards in the cycle when the parasite enters the fish. On the contrary, the observation of fluorescence in individuals for which PCR results were negative can be explained by the herbivorous diet of Euphausiacea, and consumption of microalgae whose pigments fluoresce when exposed to low wavelengths [41]. A molecular approach was thus assessed for the fast and reliable detection of Anisakidae larvae in zooplanktonic crustacean.

The first step was to constitute infected material. Since there were no natural infected crustaceans available, artificial samples were constituted. To mimic the natural infection of Euphausiacea by Anisakidae, mean weights of both organisms were obtained. The weight ratio between Anisakidae larvae (extracted from fish) and Euphausiacea individuals was between 0.8 and 2% (data not shown). Thus, artificial mixtures of 0.5 and 1% Anisakidae weight/Euphausiacea weight were built, so as to reproduce the presence of one Anisakidae larvae in one Euphausiacea. However, since no data were available about the weight of Anisakidae larvae in the crustacean host, more stringent conditions were also assessed with 0.25 and 0.1% weight ratios. Additionally, only the Wizard Genomic DNA purification kit allowed for amplification of Anisakidae DNA, even with these stringent and limited conditions.

Moreover, complex matrices such as whole individuals might lead to the presence of PCR inhibitors or nonspecific PCR amplification. DNA extraction from whole Euphausiacea leads to a mixture of DNA coming from Euphausiacea, its stomach content, organisms living on its carapace and potential Anisakidae. Greenstone *et al.* [14] and O’Rorke *et al.* [36], while studying stomach contents of crustaceans, described trouble shooting of PCR amplification from complex mixtures and/or different organisms, whereas Itoïz *et al.* [19] described the presence of PCR inhibitors in clams depending on the tissue used for the extraction. To counter these drawbacks, confirmation steps were added to the present protocol. Like in non-specific amplifications, Sanger sequencing of PCR products made it possible to confirm the specificity of the analysis. Among 30 Euphausiacea positive for Anisakidae in PCR, the presence of Anisakidae or Raphidascaridae DNA was confirmed in 22 individuals. For the eight remaining individuals, either no sequencing or non-relevant sequences were obtained, reinforcing the hypothesis of non-specific amplification. This result confirms that, at this step of method development, and before further analyses of the potential mechanisms originating the non-specific amplification, sequencing of positive results is required to ensure robust calculation of prevalence data on the basis of Sanger sequencing results. Like for PCR inhibitors, supplementary steps were carried out during protocol optimisation and/or during the sample analyses. The Wizard Genomic purification kit allowed for repeatable and systematic amplification of all tested artificial samples, even when Anisakidae DNA was present at very low concentrations in the samples (0.1% weight ratio), validating the elimination of most PCR inhibitors during the DNA extraction step. Moreover, as a supplementary precaution, during the sample analyses, each Euphausiacea DNA was tested in triplicate. Each PCR product reporting a positive result was then sequenced to confirm the results. This method was optimised and validated on Euphausiacea. It yielded very conclusive and promising results. However, even though other zooplanktonic hosts of Anisakidae are mainly crustaceans, such as Mysidacea or Copepoda, a validation step will be mandatory before extending this method to study the distribution of Anisakidae in these taxonomic groups. Naturally, to have an overall view of the circulation of Anisakidae among the zooplanktonic community, every member will have to be considered and carefully assessed for its role in this parasite lifecycle.

### Ecology of the parasite

Prevalence values observed here (2.5–4.5%) are markedly higher than those reported so far in the literature (0–4%, but usually less than 0.01%, Table 1). The higher sensitivity of the molecular method, when compared with visual observation, can be proposed as the first cause of these higher values. This would of course be consistent with the technical limitations of the visual method already mentioned and would be an advantage in favour of the PCR method. In addition, an increase in infection would also be in line with the general trend of an increase of Anisakidae infection, related to several factors, such as global warming, increases in cetaceans populations, and an increase in cetacean infection by Anisakidae [10, 33]. An increased abundance of the harbour porpoise (*Phocoena phocoena*), the most abundant cetacean species in the North Sea, has notably been observed since the early 1990s in the south of the North Sea [5]. Harbour porpoise is known to host both *Anisakis* and *Hysterothylacium* genera [26]. A recent (2009–2015) increase in *Anisakis* infection was also observed for wild salmon in Scotland [22]. Simultaneously, global warming drove a decrease in the abundance of Euphausiacea in the entire North Atlantic and also in the area studied here [8]. Euphausiacea remained, nonetheless, important in the western part of the North Sea, where it locally drives the functioning of the planktonic ecosystem. It is notably an indicator taxon of this area, and explains the difference in plankton assemblages between the west of the North Sea and the other areas (A. Delegrange unpubl. results; [35]). These simultaneous trends for both first and final hosts of Anisakidae are a reasonable explanation for increased prevalence. If the number of Anisakidae eggs emitted in the water is on the rise, as the number of cetaceans is increasing, while the number of Euphausiacea is going down, the probability of an Anisakidae larva encountering an Euphausiacea increases mathematically. Similarly, Anisakidae eggs hatch faster in warmer waters. In the context of global climate change, this also increases infection probabilities. All of these factors seem to confirm that an increase in Anisakidae infection in Euphausiacea is plausible, and that the pattern detected by the method is consistent with ecological patterns.

The species diversity of nematodes identified with sequencing is also consistent with the pattern observed for other members of the cycle, and may illustrate global climate change effects. Euphausiacea were mostly infected with *A. simplex* and, to a lesser extent, with *H. aduncum*, a pattern already described by Smith [50]. Regarding fish, *A. simplex* is commonly observed as the dominant species of Anisakidae in this area [1, 7, 11]. It dominates over *H. aduncum* in several fish species in the area, e.g. grey gurnard *Eutrigla gurnardus* [21], beaked redfish *Sebastes mentella* [23], Atlantic salmon *Salmo salar* [22], or cod *Gadus morhua* [39]. Lastly, *A. simplex* is the main Anisakidae species infecting *P. phocoena* in the North Sea [26]. The observation of one *A. pegreffii* sequence can be considered unexpected, as this species is considered to be restricted to the western Mediterranean Sea and the southern coast of the North Atlantic, i.e. off Spain and Portugal [34]. Nonetheless, some recent occurrences of *A. pegreffii* in cod [11] or Atlantic mackerel *Scomber scombrus* [27] in the North Sea, along with the observation of an *A. simplex* × *A. pegreffii* hybrid in hake *Merluccius merluccius* off Southwest Ireland [1] may challenge our understanding of the current geographical distribution of *A. pegreffii.* In these studies, low prevalence (1–9 infected fish) is interpreted as the transport of *A. pegreffii* away from its usual area of presence through host feeding migrations. However, increasing observations might illustrate a shift in the geographical distribution of this species. *A. simplex* and *A. pegreffii* have different thermic preferenda, as *A. simplex* eggs and L1 larvae are more adapted to colder waters than *A. pegreffii*’s [12]. Warming of North Sea waters might favour the achievement of *A. pegreffii* cycle, and allow some eggs of this species to hatch in these waters, a phenomenon that is likely to intensify as warming continues. More repeated analyses on all members of the life cycle would be needed to ascertain a shift northward in *A. pegreffii* distribution, and potentially relate it to warming waters. The present method would be appropriate to do so in zooplanktonic hosts.

Eventually, this preliminary sampling will provide an opportunity to continue exploring the impact of depth on Anisakidae infection. Anisakidae are well-known to be pelagic parasites. Their entire cycle implies pelagic organisms, and they have developed adaptation to the pelagic realm, like cuticles that increase the buoyancy of the second stage larvae [25]. Previous studies on fish have demonstrated higher infection at the deepest stations for individuals of the same species [7, 18, 44] or, when comparing certain species, for those with a higher ability to forage in the pelagic realm [29]. Similarly, for Euphausiacea, Smith [50] confirmed a depth effect, as he observed no infected Euphausiacea from shallow stations, but infected ones in 10 out of the 31 deep stations (>100 m depth), and with a prevalence of 0.54% (15 individuals infected out of 2 687 observed). Results obtained here are consistent with this pattern, as the prevalence is the highest for the deepest station. The work performed here was not dedicated to an actual analysis of real data, explaining why so few stations were used. However, the data appear promising and will need to be confirmed by applying this method to a larger sample. For example, spatial variation of Euphausiacea infection in the English Channel would be needed to better support the role of Euphausiacea in the infection pattern observed for horse mackerel [7]. Similarly, investigating seasonal variations in Euphausiacea infection would be required to improve our understanding of seasonal patterns in fish.
